# Evaluation of the congenital air way malformation in children with congenital heart disease using cardiac MR

**DOI:** 10.1186/1532-429X-17-S1-P217

**Published:** 2015-02-03

**Authors:** Ming Zhu

**Affiliations:** Radiology Dept, Shanghai Children’s Medical Center, Shanghai, China

## Background

The cardiovascular magnetic resonance (CMR) is usually complements echocardiography, provides a non- ionizing radiation evaluation of congenital heart disease(CHD). The congenital air way malformation occur significantly more patients with CHD than patients without CHD. Echocardiography can not show air way well and CT can show air way very well. MRI has emerged as a powerful approach to imaging CHD in children. The question is if MRI is good enough for showing air way malformation.

## Methods

We review our 428 MR cases of CHD in 2014. CMR was performed using a 1.5T unit(Achieva Nova dual; Philips). The range of age in 428 patients was 10 days to 12 years (mean 3.2 years). Imaging sequences included ECG-triggered 2D balanced steady state free precession (b-SSFP) Cine, phase contrast sequences cine (PC Cine), ECG-triggered 2D Spin echo black blood, Contrast-enhanced 3D magnetic resonance angiography (CE-MRA), Navigator-gated, ECG-triggered 3D balanced steady state free precession (SSFP) MRA and Navigator-gated, ECG-triggered 3D T1W TFE sequences. Minimum-intensity (MinIP) reconstruction was used to evaluate the tracheobronchial tree in CE-MRA, 3D SSFP and 3D T1W TFE sequences.

## Results

The tracheobronchial tree can be visualized clearly in 82.7% (286/348) of CE-MRA sequences, in 92.6% (224/242) of 3D SSFP sequences and in 98.7% (155/157) of 3D T1W TFE sequences.

In 36 of the 428(8.4%)cases with CHD, congenital air way malformation observed in MRI. The congenital air way malformation include tracheal bronchus 18, asplenia syndrome 7 (bilateral right bronchi), polysplenia syndrome 3 (bilateral left bronchi), congenital tracheal stenosis 3, bridging bronchus 3 and bilateral tracheal bronchi with asplenia syndrome 2 cases. The bilateral tracheal bronchi with asplenia syndrome is very rare congenital air way malformation.

## Conclusions

This data shows that congenital air way malformation is common in children with CHD. Our data also means that MRI also can show tracheobronchial tree and navigator-gated, ECG-triggered 3D T1W TFE sequences and MinIP reconstruction is the best sequences for air way.

